# Smoking Related Diseases: The Central Role of Monoamine Oxidase

**DOI:** 10.3390/ijerph8010136

**Published:** 2011-01-14

**Authors:** Francine Rendu, Katell Peoc’h, Ivan Berlin, Daniel Thomas, Jean-Marie Launay

**Affiliations:** 1 UMRS 956 (Génétique, Pharmacologie et Physiopathologie des maladies cardiovasculaires), Faculté de Médecine Pitié-Salpétrière, Université Pierre et Marie Curie, 91 Bd de l’hôpital, 75634 Paris cedex 13, France; 2 Service de Biochimie et Biologie Moléculaire, Assistance Publique des Hôpitaux de Paris (AP-HP), and Biologie cellulaire, Faculté de Pharmacie, Université Paris-Descartes, Hôpital Lariboisière 2 rue Ambroise Paré, 75475 Paris cedex 10, France; E-Mails: katell.peoch@lrb.aphp.fr (K.P.); jean-marie.launay@lrb.aphp.fr (J.-M.L.); 3 Pharmacologie clinique du tabagisme; Inserm U894, Faculté de Médecine Pitié-Salpétrière, Université Pierre et Marie Curie and Service de pharmacologie, Hôpital Pitié-Salpétrière, 47 Bd de l'Hôpital, 75651 Paris cedex13, France; E-Mail: ivan.berlin@psl.aphp.fr; 4 Institut de Cardiologie, Hôpital Pitié-Salpétrière, 47 Bd de l'Hôpital, 75651 Paris cedex13, France; E-Mail: daniel.thomas@psl.aphp.fr

**Keywords:** smoking, serotonin, norepinephrine, monoamine oxidase, cardiovascular, platelets, epigenetic, cancer, depression

## Abstract

Smoking is a major risk factor of morbidity and mortality. It is well established that monoamine oxidase (MAO) activity is decreased in smokers. Serotonin (5-HT), a major substrate for MAO that circulates as a reserve pool stored in platelets, is a marker of platelet activation. We recently reported that smoking durably modifies the platelet 5-HT/MAO system by inducing a demethylation of the *MAO* gene promoter resulting in high MAO protein concentration persisting more than ten years after quitting smoking. The present data enlarges the results to another MAO substrate, norepinephrine (NE), further confirming the central role of MAO in tobacco use-induced diseases. Thus, MAO could be a readily accessible and helpful marker in the risk evaluation of smoking-related diseases, from cardiovascular and pulmonary diseases to depression, anxiety and cancer. The present review implements the new finding of epigenetic regulation of *MAO* and suggests that smoking-induced *MAO* demethylation can be considered as a hallmark of smoking-related cancers similarly to other aberrant DNA methylations.

## 1. Smoking Is a Prevalent Risk Factor of Morbidity and Mortality

Tobacco use is the leading cause of preventable death and morbidity worldwide. Smoking is a modifiable risk factor for three major diseases, namely cardiovascular diseases, pulmonary diseases and cancer. This addictive disorder is responsible for one over five deaths and cardiovascular diseases is the leading cause of death from smoking. Among cardiovascular risks factors, smoking is second following dyslipidemia, with no threshold, no incidence of the length of smoking and a major prevalence among people less than 50 years for both sexes [1]. It is a major determinant of chronic obstructive pulmonary disease (COPD) and the most important risk factor of lung cancer.

The molecular mechanisms underlying smoking-induced diseases are not fully understood, although all have in common systemic inflammation and oxidative stress. While nicotine is considered to be the major player underlying tobacco dependence [2], many other tobacco smoke constituents are also involved in smoking-induced dependence and diseases, including additives to enhance the addictiveness [3–5]. Another target of smoking is obviously the mitochondria whose most fundamental function is the production of ATP in the cellular respiratory chain. Of the very few oxygen-dependent enzymes (aromatic amino acid hydroxylases and nitric oxide synthases for instance), the mitochondrial MAO have been shown to be impacted by smoking [6–8].

## 2. MAO and Smoking

MAO (EC 1.4.3.4) are integral proteins of outer mitochondrial membranes devoted to the organism homeostasis protection. They are major enzymes which occur in various cells, both neuronal and non-neuronal in the central nervous system (CNS) and peripheral organs. In the CNS, MAO not only plays a physiological role in the metabolic inactivation of released monoamine transmitters (catecholamines, 5-HT) and in the detoxification of xenobiotic amines but also a pathophysiological role by generating cytotoxic free radicals during aging and neurodegenerative diseases. These enzymes are abundant in organs in direct relation with the environment: lungs, gastrointestinal tractus and liver. Their main function is to proceed an oxidative deamination of biogenic amines, both exogenous (tyramine) and endogenous (NE, dopamine and 5-HT) in peripheral tissues and brain. Thus, MAO enzymes are involved in the regulation of neurotransmitters which control mood, behaviour, movement, memory, appetite, sleep and personality. Their inhibition, by either exogenous or pharmaceutical substances, leads to the increase of the above monoamines and has major consequences in the central nervous system.

The first report that MAO activity is modified by smoking appeared in the early eighties in a mouse model [6] and in human cigarettes smokers [7]. It is only fifteen years later that Berlin *et al.* [8] measured lower activities of MAO in heavy smokers. Studies using PET-scan showed that this inhibition occurs both in smokers’ peripheral tissues and different organs, lungs, kidneys, brain and spleen [9,10]. The reduction in brain MAO-B is widespread and the degree of inhibition is of the same order of magnitude than that found in platelets of current smokers. Such a large distribution of MAO accounts for the implication of these enzymes in cardiovascular diseases, pulmonary diseases, cancer and affective spectrum disorders.

There are two MAO isoenzymes, MAO-A and MAO-B, which differ by their substrate specificity and even more by inhibitor selectivity. MAO-A is inhibited by low concentrations of clorgyline whereas MAO-B is irreversibly inhibited by low concentrations of deprenyl. In humans, the highest concentrations of MAO-A are found at the organism’s barrier: gut, placenta, lungs and liver, corresponding to the enzyme’s ontogenetic detoxifying function. High concentrations of MAO-B are found in glial cells and blood platelets. MAO-A preferentially deaminates bioamines of endogenous source such as 5-HT, epinephrine and NE, while MAO-B preferentially degrades exogenous bioamines ingested in the diet such as phenylethylamine and benzylamine [5,11]. Dopamine and tyramine are equally catabolized by both forms of MAO. These substrate specificities are relative, however, since, depending on conditions, all the above bioamines are substrates for both isoenzymes. End products of the MAO action on bioamines are aldehydes and H_2_O_2_ involved in oxidative processes. Aldehydes are either oxidized in 5-hydroxy indol acetic acid (5-HIAA) for 5-HT catabolism or reduced in 3,4-dihydroxyphenylglycol (DHPG) for NE catabolism. The formation of 5-HIAA from 5-HT via MAO is accompanied by the generation of ROS (see [5] for a review) which participate in smooth muscle cell proliferation, hypoxia and respiratory distress [12–14] and cancer growth [15].

The genes coding for MAO-A and MAO-B are both located on the short arm of the X chromosome [16]. Comparison of the deduced amino-acid sequences showed that MAO-A and MAO-B have around 70% amino-acid sequence identity. The two genes *MAOA* and *MAOB* are arranged in a tail-to-tail orientation, and both span at least 60 kb, consist of 15 exons, and exhibit an identical exon-intron organization [17]. The promoter regions share 60% sequence homology and both promoters consist of GC-rich regions. Within the *MAOA* promoter region, a polymorphism has been shown to affect expression [18] and, using bioinformatics approaches, putative methylation targets have been identified [19]. The repeat 3 of VNTR polymorphism of *MAOB* intron 13 has also been implied into the men smoking predisposition when associated with the dopamine D2 receptor gene (*DRD2*) polymorphism [20]. An association between the 4-repeat allele of MAO-A functional VNTR promoter polymorphism and the tobacco consumption was also evidenced in caucasian male patients [21]. Altogether, although still unclear, there is some evidence that *MAO* genetic factors could favour tobacco addiction and the resulting biogenic amine modulation.

## 3. Bioamines Catabolism in Smokers

It is well established that smoking modifies MAO, but until recently, it was not clearly stated whether it is the enzyme amount or its activity which is smoking sensitive. In most of reports, it has been evaluated by PET [9,10,22]. The PET scan analysis of MAO allows to (i) mainly visualize the enzyme location and (ii) appreciate its concentration by a measure of the color deduced from the autoradiographic density. It does not allow, however, an accurate measure of the enzyme amount. The latter also applies to the Western blot technique, which allows an estimation of MAO, but again quantitation of the band intensity is only approximate. The only real quantitative assessment is a radiochemical assay based upon the binding of a MAO reversible inhibitor, as developed by Cesura *et al.* [23]. Such a technical point is of peculiar importance when accurate comparisons of MAO protein levels have to be performed, *i.e.*, for instance between smokers and former smokers.

With the aim to clarify whether tobacco use affects MAO activity and/or MAO amount, we used the biochemical assay described by Cesura *et al.* [23] and took advantage of the easy access to the MAO-B isoform contained in blood platelets to measure both its amount and its activity [24]. Following careful pre-analytical conditions for blood sampling, two major MAO substrates, 5-HT and NE, were measured in blood platelets and in plasma of 115 men, respectively. Technical details can be found in references [8] and [24].

### 3.1. 5-HT and 5-HIAA

It has been proposed that 5-HT could be predictive of coronary artery diseases, especially in young people [25]. In the latter report, it was not clear, however, which 5-HT pool was quantified, *i.e.*, the platelet stored content or the plasma pool. This is of importance, since platelet 5-HT storage prevents 5-HT-induced vascular effects. Platelet activation is associated with both progression of atherosclerosis and precipitating events leading to stenosed arteries [26,27]. Activated platelets release their granule content which leads to the exposition of new adhesive receptors and secretion of cytokines, RANTES and growth factors [28–30]. Among these released growth factors, serotonin is quantitatively the major one considering its millimolar concentration within platelet dense granules. High plasma serotonin contributes to activate lung vascular receptors [31,32] and pulmonary hypertensive complications are observed [33]. In *in vitro* experiments, serotonin induces smooth muscle cell proliferation [34,35] and contributes to inflammatory and thrombotic activation of the vessels [36]. In atherosclerotic mice, platelet serotonin granule secretion plays a critical role in vascular remodelling [37]. And so, serotonin receptor blockers are potent vasodilators in various cardiovascular diseases [38] and the serotonin receptor 5-HT_2A_ antagonist ketanserin contributes to reduce neointimal proliferation [39].

In this context and considering that: (i) smoker’s platelets are activated [30,40] and (ii) cigarette smoke directly causes platelet activation [41], we postulated that in smokers’ platelets the granule-stored 5-HT would be released and degraded by MAO. We thus performed a comparison between non smokers, smokers and former smokers of 5-HT and its catabolite 5-HIAA measured in isolated blood platelets and in plasma, respectively. Contrary to all expectations, platelet 5-HT was not found different in smokers and non-smokers but was lower in former smokers with a parallel higher plasma level of 5-HIAA [24]. Thus, our precise determination of platelet 5-HT content and plasma 5-HIAA did not confirm that 5-HT could be a cardiovascular risk marker [25]: if platelet 5-HT and plasma 5-HIAA levels were both correlated with the cardiovascular risk reference—the Framingham risk score [24], after adjusting these correlations to the smoking status, it appeared that the determining risk factor among those taken into account in the Framingham risk calculation was smoking.

**Figure 1:**
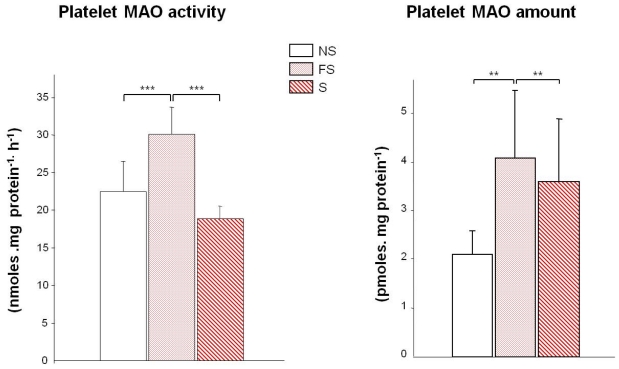
Platelet MAO in smokers (44 S), non smokers (34 NS) and former smokers (37 FS). Left: MAO activity (nmoles.mg protein^−1^h^−1^) Right: MAO amount (pmoles.mg protein^−1^). **P < 0.001, ***P < 0.00001.

The “apparently normal” level of 5-HT in smokers’ platelets was unforeseen since MAO-B activity is inhibited in smokers [8–10,22,24]. The quantitative measure of MAO amount rendered our results coherent (Figure 1): in smokers and former smokers the smoking-induced inhibition of MAO activity is counter-balanced by higher enzyme amounts. After quitting smoking, MAO activity is no longer inhibited but the high amount of protein results in a higher 5-HT degradation into 5-HIAA. The MAO activity did not correlate with any studied marker other than platelet 5-HT and plasma 5-HIAA (P < 0.0001). The most striking results is that the high smoking-induced platelet MAO-B amount remains elevated several years after quitting smoking (mean 13 years). Further studies are needed, however, to: (i) determine the delay between the smoking start and the elevation of MAO-B protein concentration and (ii) to precisely establish how long after quitting smoking this effect lasts. Preliminary unpublished data suggest a reversion of this increase of protein synthesis around 20 years after quitting smoking.

### 3.2. NE and DHPG

**Figure 2:**
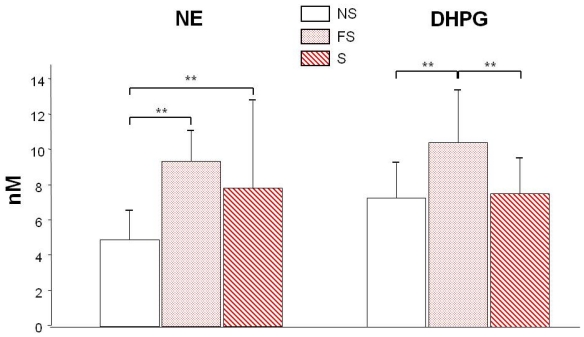
Norepinephrine (NE, left) and its metabolite DHPG (right) in the plasma from non smokers (white bars), former smokers (FS dotted bars), and smokers (S hatched bars) **P < 0.001; Differences between FS and S were not significant (*P* > 0.05) for NE and the same applies between NS and S for DHPG. NE and DHPG were determined by HPLC with electrochemical detection as previously described [8].

Because platelet NE amount is high, we also measured NE and DHPG in carefully drawn and processed plasma samples. Deamination of NE by MAO produces a reactive aldehyde that is reduced to form DHPG, a minor but MAO-specific NE catabolite. The highest NE plasma level was found in former smokers especially compared to never smokers (Figure 2 left). Fitting with the high level of plasma NE, its catabolite, DHPG, was higher in former smokers’ plasma as well (Figure 2 right). However, the DHPG/NE ratio was significantly (*P* < 0.01) lower for smokers (1.40 *vs.* 1.72 and 1.79 for never smokers and former smokers, respectively) indicating a reduced MAO-A activity in smokers without persistence in former smokers. Like platelet 5-HT, the plasma level of NE significantly correlated with the MAO protein amount (*P* < 0.0001). Low platelet contents of 5-HT and high plasma levels of NE in former smokers are consistent with a high MAO amount of enzyme active after quitting smoking [24]. Moreover, as for platelet 5-HT, plasma NE correlated with the length of smoking (p < 0.0009).

## 4. Is MAO a Risk Marker?

**Figure 3:**
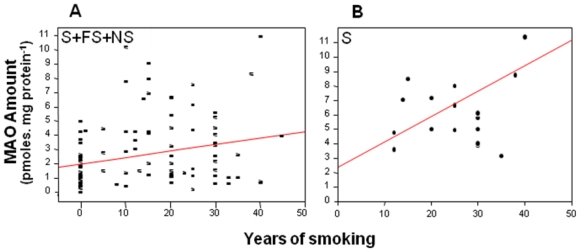
Correlation between platelet MAO amount and duration of smoking. A: in the whole population (smokers S + former smokers FS + non smokers NS: n = 108, r = 0.25, *P* < 0.01) and B: in smokers (S) above the cut-off value (n = 18, r = 0.77, *P* < 0.01).

Among the population studied, the MAO-B amount correlates significantly with the number of atherosclerotic plaques, the number of atherosclerotic sites, especially the femoral sites [24]. It also correlated with the duration of smoking (Figure 3 left), indicating that the MAO-B protein concentration is linked to the duration of smoking. At this point, an interesting observation comes to light. The median value of the MAO amount in our whole population was of 2.5 pmoles/mg platelet protein. For those for which MAO amount was over the median value, considered as a cut off value, it significantly correlated with the length of smoking (not shown): the longest time they smoked, the higher amount of MAO they had. By contrast, for those with less MAO than the cut off median value, there was no correlation with the duration of smoking. This holds also true for smokers. As illustrated on (Figure 3 right), only a MAO amount higher than 2.5 pmoles/mg platelet protein was correlated (*P* < 0.01) to the duration of smoking. This observation suggests that the duration of smoking has no incidence provided the MAO amount before smoking is less than 2.5 pmoles/mg platelet protein. Furthermore, whereas only 27% of non smokers have a MAO concentration over 2.5 pmoles/mg platelet protein, it comes to 45% of smokers and 77% of former smokers. Altogether, this observation suggests that MAO-B amount could well be a morbidity determinant. Obviously this point should be studied in a larger population in order to really establish whether there may be a critical level of MAO concentration around 2.5 pmoles/mg platelet protein above which morbidity risk would be higher.

## 5. Smoking-induced Epigenetic Modification of MAO

**Figure 4:**
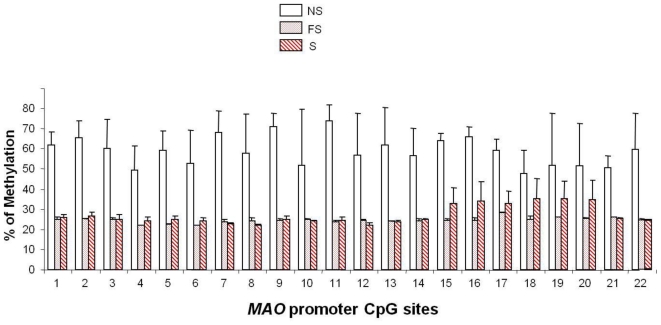
Methylation of the 22 CpG sites of the *MAOB* promoter in PBMC from non smokers (4 NS, white bars), former smokers (4 FS, dotted bars) and smokers (5 S, hatched bars). The differences between NS and FS or S are significant at all sites (0.0001 < *P* < 0.02) except at sites 19–20.

A persistent increase in the amount of MAO-B lasting over 10 years after quitting smoking suggested a modification at the gene level. Indeed the smoking-induced variations of MAO expression are explained by a reduced methylation level of the *MAOB* promoter [24] (Figure 4), resulting in a more active transcription of the *MAO* and hence a higher MAO amount. The higher the *MAOB* promoter is methylated, the lower MAO amount is found. Human *MAOB* core promoter comprises 22 CpG sites that can be methylated, with methylation reducing the transcription [42]. The extent of methylation of the *MAOB* promoter is markedly lower at almost all CpG methylation sites for smokers and former smokers compared to non smokers (from 59% to 25 and 27%, respectively), except sites n°19 and 20. Moreover, the MAO level was positively correlated to the methylation extent of all sites (0.0002 < *P* < 0.003) except that of site n°19. The study of a much larger series, using high-throughput technologies allowing more rapid direct measures of DNA methylation, would enable a more precise and detailed analysis. Such a study would allow defining if there are specific sites which are more determinant for smoking-induced risks through impact on MAO amount.

How does such a DNA methylation occur? Based on experiments performed on an *ex vivo* animal model we proposed that tobacco smoke induces an increase of nucleic acid demethylase activity [24], possibly due to hyperactive poly (ADP-ribose) polymerases [43,44]. This may not apply to humans, however, because species differences in the enzymatic process cannot be excluded. Besides, since no DNA demethylase enzyme has yet been identified, it could also result from a reversion of the transmethylase(s).

## 6. MAO in Smokers: From Cardiovascular Diseases to Mood Disorders

Several data indicate that smoking is an anti-depressive addict (see [5] for a review]. Moreover, keeping in mind that for many years the prevailing hypothesis on depression is an absolute or relative deficiency of monoamines [45], the differences measured between smokers and non smokers could have implication in their respective behaviours. Despite the participants to our study were clearly not addicts since they came for an evaluation of their cardiovascular risk, about half of the participants were submitted to an anxiety-depression questionnaire (HAD test). Among the 68 participants, only 5% had scores above the characteristic threshold for depression and anxiety, all of which were smokers. An obvious gradient from smokers (mean values of 4.5 and 7.6), to never smokers (3.2 and 6) and former smokers (2.3 and 6) appeared for the tendency to depression and anxiety, respectively. In agreement with previous reports, the smoker group was the most proned to depression. One can therefore speculate that smokers need to inhibit their MAO by smoking instead of using a MAO inhibitor, the first class of antidepressants. As for the group of former smokers, not only they were the less depressed, but they were in a more euphoric mood than non smokers. This is to put together with the higher plasma levels of NE of former smokers, since catecholamines are crucial for affective disorders.

## 7. Conclusions

This review summarizes our recent finding that smoking durably reduces the methylation of the *MAOB* promoter, leading to an increase in MAO-B protein synthesis. In other words, smoking provokes a higher MAO-B synthesis to compensate the decreased enzyme activity due to oxygen deprivation. Despite the fact that both MAO-B and MAO-A activities are affected by smoking, it is important to underscore that the two MAO isoforms and the two unique genes encoding these enzymes, share some common characteristics but also have unique features. In smoking women a similar approach would be of interest to explore a possible gender specificity [46]. If similar epigenetic modification(s) upon one or both MAO isoform(s) are found in women, they may account—at least partially—for transgenerational consequences following *in utero* exposure to maternal smoking [47]. We also extended our results to point out that MAO-B could be a risk biomarker of smoking-induced morbidity, due to the role of the bioamines/MAO system in all smoking related diseases. This risk factor can easily be measured from a blood platelet sample. Its cut-off can be estimated at around 2.5 pmoles/mg platelet protein, but has now to be precisely refined in larger series.

A holistic approach to the smoking related diseases, *i.e.*, cardiovascular diseases, pulmonary diseases, lung cancer, and mood disorders may be to further study the role of MAOs in these diseases. Smoking is the best example of lifestyle influence which considerably modifies the epigenetic patterns in various tissues (see [48] for a review] and MAOs might be a determinant component to explore the exposome [49]. Hopefully, epigenetic modifications are reversible and our preliminary results indicate that the decrease in DNA methylation is back to normal around twenty years after quitting. The potential value of MAO-B as a smoking biomarker might therefore be of public health relevance to help in individual susceptibility evaluation and disease diagnosis and prognosis.
